# Hemotropic mycoplasmas in little brown bats (*Myotis lucifugus*)

**DOI:** 10.1186/1756-3305-7-117

**Published:** 2014-03-24

**Authors:** Patricia E Mascarelli, Michael K Keel, Michael Yabsley, Lisa A Last, Edward B Breitschwerdt, Ricardo G Maggi

**Affiliations:** 1 College of Veterinary Medicine, North Carolina State University, 1060 William Moore Drive, Raleigh, NC 27607, USA; 2 Department of Population Health, Southeastern Cooperative Wildlife Disease Study, College of Veterinary Medicine, University of Georgia, Athens, GA 30602, USA; 3 Department of Pathology, Microbiology, and Immunology, University of California, Davis, CA 95616, USA; 4 D.B. Warnell School of Forestry and Natural Resources, University of Georgia, Athens, GA 30602, USA

**Keywords:** Hemotropic mycoplasma, Bat, Haemoplasma, Mycplasma, WNS

## Abstract

**Background:**

Hemotropic mycoplasmas are epicellular erythrocytic bacteria that can cause infectious anemia in some mammalian species. Worldwide, hemotropic mycoplasmas are emerging or re-emerging zoonotic pathogens potentially causing serious and significant health problems in wildlife. The objective of this study was to determine the molecular prevalence of hemotropic *Mycoplasma* species in little brown bats (*Myotis lucifugus*) with and without *Pseudogymnoascus* (*Geomyces*) *destrucans*, the causative agent of white nose syndrome (WNS) that causes significant mortality events in bats.

**Methods:**

In order to establish the prevalence of hemotropic *Mycoplasma* species in a population of 68 little brown bats (*Myotis lucifugus*) with (n = 53) and without (n = 15) white-nose syndrome (WNS), PCR was performed targeting the 16S rRNA gene.

**Results:**

The overall prevalence of hemotropic Mycoplasmas in bats was 47%, with similar (p = 0.5725) prevalence between bats with WNS (49%) and without WNS (40%). 16S rDNA sequence analysis (~1,200 bp) supports the presence of a novel hemotropic *Mycoplasma* species with 91.75% sequence homology with *Mycoplasma haemomuris*. No differences were found in gene sequences generated from WNS and non-WNS animals.

**Conclusions:**

Gene sequences generated from WNS and non-WNS animals suggest that little brown bats could serve as a natural reservoir for this potentially novel *Mycoplasma* species. Currently, there is minimal information about the prevalence, host-specificity, or the route of transmission of hemotropic *Mycoplasma* spp. among bats. Finally, the potential role of hemotropic *Mycoplasma* spp. as co-factors in the development of disease manifestations in bats, including WNS in *Myotis lucifugus*, remains to be elucidated.

## Background

Hemotropic mycoplasmas (hemoplasmas, formerly classified as *Haemobartonella* and *Eperythrozoon* spp.), are epicellular erythrocytic bacterial parasites lacking a cell wall, that can cause infectious anemia in some mammalian species [1-5]. Worldwide, hemotropic Mycoplasmas are emerging or re-emerging zoonotic pathogens that affect livestock [6-14], wildlife [15-19], companion animals [4,20-27], and humans [28-34]. These bacteria can cause serious and economically significant health problems in production animals. Infections with hemotropic Mycoplasmas can range from asymptomatic to illnesses characterized by overt life-threatening hemolytic anemia, subtle chronic anemia, ill-thrift, and infertility. In addition, these cell wall deficient bacteria may act as cofactors in the progression of retroviral, neoplastic, and immune-mediated diseases [1,34,35]. Unfortunately, little is known about hemotropic *Mycoplasma* spp. prevalence, host-specificity, or route of transmission in many wildlife species.

Historically, the diagnosis of hemotropic *Mycoplasma* infections relied upon cytological examination of stained blood smears. However, diagnostic sensitivity of blood smear examination is generally less than 20%, and specificity is hampered by artifacts, such as stain precipitates and Howell-Jolly bodies [23,36,37]. The development of molecular assays, primarily targeting the 16S rRNA gene of these microbes, has resulted in recognition of several novel animal hemotropic mycoplasmas [37-39], hence the host range has increased in recent years.

The objective of this study was to determine the molecular prevalence of hemotropic *Mycoplasma* species in a population of 68 little brown bats (*Myotis lucifugus*) from Northeastern and Eastern US. In addition, all bats were tested for *Pseudogymnoascus* (*Geomyces*) *destructans*, the causative agent of white nose syndrome (WNS) and cause of significant mortality events in bats, to determine if there was an association with *Mycoplasma* infection status.

## Methods

### Sample collection

A total of 68 dead little brown bats were sampled primarily during the mid-hibernation period from eastern and northeastern US (Pennsylvania, Ohio, Kentucky, West Virginia, Tennessee and North Carolina). Protocols for capturing, handling and sample collection followed the United States Fish and Wildlife Service Disinfection Protocol for Bat Studies. Dead bats collected by hand from roost substrates, were individually placed in plastic bags and stored at −20°C until processing. Each bat was submitted to the Southeastern Cooperative Wildlife Disease Study at the College of Veterinary Medicine, University of Georgia, Athens, Georgia, where gross examination was carried out on carcasses. To avoid DNA cross-contamination, expendable supplies were used for each animal. Bat samples were tested for *P. destructans* by histological examination and molecular testing, either targeting the internal transcribed spacer (ITS) region of the rRNA gene complex [40,41] or the intergenic spacer (IGS) region [42,43], as previously reported. Tissue samples (spleen) collected from each individual were placed in 70% ethanol and stored at −80°C until DNA extraction and molecular analysis for evidence of hemotropic *Mycoplasma* infections were performed.

### Nucleic acid preparations

Total DNA from 25 mg of spleen tissue from each bat was extracted according to manufacturers instructions using a QIAamp DNA Mini Kit^a^. After extraction, DNA concentration and quality was measured using absorbance ratio between 260/280 nm^b^. DNA was stored at −20°C until testing.

### PCR amplification

Amplification of hemotropic *Mycoplasma* 16S rDNA was performed using two sets of oligonucleotides as previously described [17]: HemMycop16S-41 s: 5’ GYA TGC MTA AYA CAT GCA AGT CGA RCG 3’ and HemMyco16S-938as: 5’ CTC CAC CAC TTG TTC AGG TCC CCG TC 3’ and HemMycop16S-322 s: 5’ GCC CAT ATT CCT ACG GGA AGC AGC AGT 3’ and HemMycop16S- 1420as: 5’ GTT TGA CGG GCG GTG TGT ACA AGA CC 3’. Sequences derived from amplicons obtained from each primer set (with an overlap of 600 bp) were aligned and edited using AlignX (Vector NTI suite 11.5.1). Amplification was performed in a 25-μl final volume reaction, the 25 μL reaction mix contained 12.5 μL of Takara Ex Taq DNA Polymerase® Premix (Fisher Scientific, Hampton, NH, USA), 0.2 μL of 100 μM of each forward and reverse primer, 7.3 μl of molecular grade water and 5 μl of template DNA. Five microliters of RNAse free water was used as a PCR negative control. Positive controls were prepared using 5 μl of DNA from dog blood spiked with a 700 bp region of *M. hematoparvum* 16S rRNA cloned in pGEM plasmid at a final concentration of 2 copies per microliter. Amplification was performed in an Eppendorf Mastercycler EPgradient® (Hauppauge, NY, USA) as previously described [17]. PCR products were analyzed by 2% agarose gel electrophoresis and detected using ethidium bromide under ultraviolet light. Amplicon products were sequenced by Eton Bio, Inc^.^ (RTP, NC, USA) to establish species strain identification using chromatogram and alignment analysis (ContigExpress® and AlignX software, Vector NTI® v10, Invitrogen, Carlsbad, CA, USA).

### Phylogenetic analysis

Each 16S rRNA sequence was compared to 26 other hemotropic *Mycoplasma* sequences deposited in GenBank database in order to compare phylogenetic relatedness (evolutionary history) using the Neighbor-Joining method (MEGA4® software). The tree is drawn to scale, with branch lengths in the same units as those of the evolutionary distances used to infer the phylogenetic tree. The evolutionary distances were computed using the Maximum Composite Likelihood method and are in the units of the number of base substitutions per site. MEGA4.

## Results

### Molecular and histological examination of little brown bats

From a total of 68 bats, 53 (78%) had gross and histologic lesions consistent with WNS and were all PCR positive for *P. destructans*. The remaining 15 bats did not show any signs of clinical abnormalities or fungal infection.

### Hemotropic mycoplasma spp. DNA analysis

A total of 32 (47%) bats tested positive for hemotropic *Mycoplasma* spp. using primers targeting a region covering 700 bp of the 16S rRNA gene. All 32 sequences were identical. Interestingly, a similar prevalence was found for hemotropic *Mycoplasma* infection in bats with and without WNS (26/53 (49%) and 6/15 (40%) respectively). Mycoplasma amplification did not occur in 27 and 9 bats with and without evidence of WNS, respectively. There was no statistical difference (Fisher’s exact, p = 0.5725) between the prevalence of hemotropic *Mycoplasmas* in WNS vs non-WNS affected bats.

**Figure 1:**
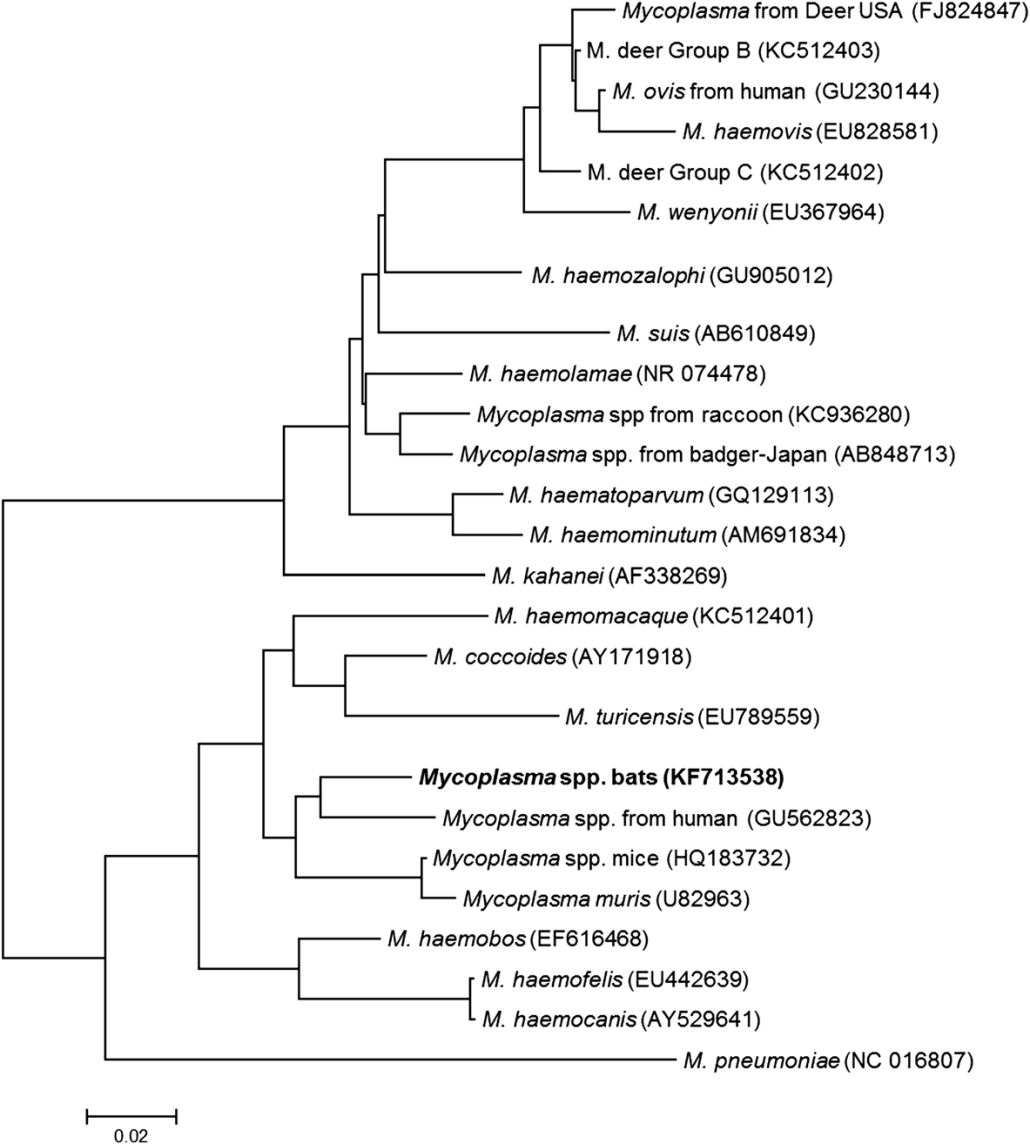
**Neighborhood-joining analysis using 25 taxa of hemotropic *Mycoplasma *species (including *Mycoplasma pneumonia*) from the Genbank database and the 16S rRNA gene sequence obtained from the brown bats (Genbank accession KF713538) in this study.** Candidatus status was omitted for simplicity. The evolutionary relationship was inferred using the Neighbor-Joining method. The optimal tree with the sum of branch length = 0.95954352 is shown. The tree is drawn to scale, with branch lengths in the same units as those of the evolutionary distances used to infer the phylogenetic tree. The evolutionary distances were computed using the Maximum Composite Likelihood method and are in the units of the number of base substitutions per site. All positions containing gaps and missing data were eliminated from the dataset (Complete deletion option). Phylogenetic analyses were conducted in MEGA4.

A longer DNA sequence was obtained for 18 randomly selected PCR positive samples (four of which were from non-WNS animals), covering a 1,200 bp of the 16S rRNA gene as previously described [19]. Again, all sequences were identical. Sequence analysis using 1103/1200 bp, identified the closest homology (91.9%) to a hemotropic *Mycoplasma* detected in a human (Genbank GU562823), and 1101/1200 (91.8%) with *Mycoplasma haemomuris* (AB758440), suggesting the presence a novel hemotropic *Mycoplasma* species in the sampled little brown bats (Figure 1). There were no differences in the *Mycoplasma* 16S rDNA sequences derived from WNS-positive and WNS-negative animals. The 16SrRNA sequence of the hemotropic *Mycoplasma* species detected in bats was deposited in Genbank (accession number KF713538).

## Discussion

Hemotropic *Mycoplasma* spp. appear to have co-evolved with many animal species. The development of molecular assays, primarily targeting the 16S rRNA gene of these microbes, has resulted in the recent recognition of several novel animal and human hemoplasmas [15,17-19,44-47]. This study represents the first report of a novel, and as yet incompletely characterized hemotropic *Mycoplasma* species in little brown bats, with an overall prevalence of 47%. There was no causative association with WNS, suggesting that this bat species acts as a natural reservoir for this uncharacterized *Mycoplasma* species. It is important to note that the results presented here may be biased either by the low number of bats assessed in either group or the lack of appropriate control bats obtained from non-WNS study sites. Therefore, conclusions on the role of hemotropic *Mycoplasma* as a potential co-factor in the development of WNS in bats cannot be derived from this study.

## Conclusion

The relative high hemotropic Mycoplasmas DNA prevalence detected in WNS and non-WNS animals (49% and 40% respectively) together with the sequence analysis generated from the 16SrRNA gene suggest that the little brown bats could serve as a natural reservoir for a novel hemotropic *Mycoplasma* species. Hemotropic *Mycoplasma* infection in mammals can cause a wide range of clinical conditions, from sub-clinical to life-threatening hemolytic anemia (particularly when immunosuppressed, stressed from poor nutrition, pregnancy, or lactation, or when concurrently infected with other more virulent pathogens, or more than one *Mycoplasma* species) [1,27,48]. Currently, there is no information about the prevalence, host-specificity, or the route of transmission of hemotropic *Mycoplasma* spp. in bats. The potential role of hemotropic *Mycoplasma* as a cause of disease manifestations, and specifically WNS in *Myotis lucifugus* remains to be elucidated.

## Endnotes

^a^QIAGEN Inc., Valencia, CA.

^b^Nanodrop, Thermo Scientific, USA.

## Abbreviations

WNS: White nose syndrome; RTP: Research Triangle Park.

## Competing interests

The authors declare that they have no competing interests.

## Authors’ contributions

PEM, RGM, and LAL performed the PCR testing of the patient samples, performed DNA sequencing and alignments. MKK and LAL assisted in sample acquisition and testing. EBB, MY, and RGM coordinated various aspects of the investigation. EBB, PEM, and RGM helped to draft the final manuscript. All authors read and approved the manuscript.
